# Supplementary material for: In situ evidence of thermally induced rock breakdown widespread on Bennu’s surface
Source: Nat Commun. 2020 Jun 9;11:2913. doi: 10.1038/s41467-020-16528-7 (PMC7283247; doi:10.1038/s41467-020-16528-7)
Supplement: Supplementary file 1 — Supplementary Information [file 41467_2020_16528_MOESM1_ESM.pdf]

SUPPLEMENTARY INFORMATION

In situ evidence of thermally induced rock breakdown widespread on Bennu's surface

J. L. Molaro (1), K.J. Walsh (2), E.R. Jawin (3), R.-L. Ballouz (4), C. A. Bennett (4), D. N. DellaGiustina (4), D. R. Golish (4), C. Drouet d'Aubigny (4), B. Rizk (4), S. R. Schwartz (4), R.D. Hanna (5), S. J. Martel (6), M. Pajola (7), H. Campins (8), A. J. Ryan (4), W. F. Bottke (2), and D. S. Lauretta. (1) Planetary Science Institute, Tucson, AZ USA (jmolaro@psi.edu); (2) Southwest Research Institute, Boulder, CO USA; (3) Department of Mineral Sciences, National Museum of Natural History, Smithsonian Institution, Washington D.C. USA; (4) Lunar and Planetary Laboratory, University of Arizona, Tucson, AZ USA; (5) Department of Geological Sciences, Jackson School of Geosciences, University of Texas, Austin, TX USA; (6) Department of Earth Sciences, School of Ocean and Earth Science and Technology, University of Hawai'i at Mānoa, Honolulu, HI USA; (7) INAF-Astronomical Observatory of Padova, Vic. Osservatorio 5, 35122 Padova, Italy; (8) Department of Physics, University of Central Florida, Orlando, FL 32816, USA;

Contents: Supplementary Table 1

Supplementary Table 1. Source data from the model for Figures 4 and 6

Boulder Diameter (m)	Stress (dense) (MPa)	Uncertainty (dense) (MPa)	Stress (porous) (MPa)	Uncertainty (porous) (MPa)	Temperature Amplitude (dense) (K)	Temperature Amplitude (porous) (K)
0.2	1.33	0.13	1.61	0.16	59.78	114.34
0.3	2.00	0.21	1.90	0.19	58.29	107.41
0.4	2.57	0.26	1.90	0.19	54.94	104.66
0.5	2.93	0.30	1.82	0.18	52.08	103.38
0.6	3.08	0.32	1.72	0.18	50.44	101.82
0.8	3.07	0.33	1.49	0.28	49.19	102.06
1.0	2.96	0.44	1.33	0.29	49.13	103.15
1.5	2.47	0.25	1.15	0.11	48.79	103.91
2.0	2.02	0.49	0.87	0.23	48.67	102.93
3.0	1.26	0.65	0.47	0.78	48.26	103.47
4.0	1.28	0.29	0.41	0.70	48.09	103.83
5.0	1.18	0.42	0.36	0.50	47.83	101.32
6.0	1.02	0.47	0.37	1.68	47.68	105.26
